# Physical Ergonomic Assessment in Cleaning Hospital Operating Rooms Based on Inertial Measurement Units

**DOI:** 10.3390/bioengineering11020154

**Published:** 2024-02-03

**Authors:** Daniel Koskas, Nicolas Vignais

**Affiliations:** 1 CIAMS, Université Paris-Saclay, 91405 Orsay, France; daniel.koskas@ucdconnect.ie; 2 CIAMS, Université d’Orléans, 45067 Orléans, France

**Keywords:** musculoskeletal disorders, inertial measurement units, physical ergonomic assessment, rapid upper limb assessment

## Abstract

Workers involved in hospital operating room cleaning face numerous constraints that may lead to musculoskeletal disorders. This study aimed to perform physical ergonomic assessments on hospital staff by combining a continuous assessment (RULA) based on inertial measurement units with video coding. Eight participants performed cleaning tasks while wearing IMUs and being video recorded. A subjective evaluation was performed through the Nordic questionnaire. Global RULA scores equaled 4.21 ± 1.15 and 4.19 ± 1.20 for the right and left sides, respectively, spending most of the time in the RULA range of 3–4 (right: 63.54 ± 31.59%; left: 64.33 ± 32.33%). Elbows and lower arms were the most exposed upper body areas with the highest percentages of time spent over a risky threshold (right: 86.69 ± 27.27%; left: 91.70 ± 29.07%). The subtask analysis identified ‘operating table moving’, ‘stretcher moving’, and ‘trolley moving’ as the riskiest subtasks. Thus, this method allowed an extensive ergonomic analysis, highlighting both risky anatomical areas and subtasks that need to be reconsidered.

## 1. Introduction

The COVID-19 crisis has brought to light the involvement of hospital staff and the difficulty of their work. These workers are indeed facing numerous constraints, particularly at the physical level. These constraints may contribute to different risk factors, like force exertion and awkward postures, of musculoskeletal disorders (MSDs) [1].

MSDs represent the majority of occupational diseases worldwide, i.e., all health damages that progressively occur among workers after a latency period in the course of their work [2]. They may be due to various factors such as gesture repetitiveness, force exertion, temporal pressure, awkward postures, and/or inadequate equipment. MSDs also generate socio-economic consequences at both individual and collective levels. In fact, while consequences at the individual level are functional disabilities of the worker, those at the corporate level are direct and indirect costs. The former is, for example, financial compensation for a worker, and the latter can be a decrease in productivity due to the absence or limitation of the worker [3]. In 2017, direct costs were worth two billion euros in France [4].

MSD consequences are particularly severe among hospital staff. Indeed, qualitative and quantitative studies have reported upper body pains and injuries among nurses [1,5,6], nursing assistants [7], and surgeons [8,9], with various risk factors. Common impacts are fatigue, sleeping disturbances caused by pain, and motion restrictions [6,7]. When measures are taken to relieve pain, taking days off appears to be a last-resort solution because workers do not want to burden their colleagues through their absence [7]. Although very few studies focused on hospital cleaners, they appear particularly at risk with frequent symptoms of MSDs, reduced working abilities and absenteeism due to musculoskeletal complaints [10]. Anatomical areas concerned with MSDs in hospital cleaners are the back, neck and upper limbs [11].

Although the causes of MSDs are multifactorial, physical risk factors usually prevail. Thus, a way to prevent MSDs, from a physical ergonomic perspective, is to identify risk factors through specific assessment in order to reduce their influence. Different methods and tools exist, being classified into three main families: self-reports, observational methods and direct measurements [12,13]. In recent years, combining automatized observational methods with direct measurement tools appeared to be useful for continuous assessment in real conditions. As an example, data from inertial measurement units (IMUs) fixed on the worker’s body may be entered into the rapid upper limb assessment (RULA) method in real-time for ergonomic assessment [14]. Although the combination of the RULA method with direct measurement tools has already been applied to surgeons [8,15], other hospital workers might benefit from this biomechanical approach to physical ergonomics. Previous studies focused more on quasi-static tasks, but combining a continuous recording of movement, e.g., a markerless motion capture system, to a RULA-based ergonomic assessment has also been applied to dynamic tasks [16,17].

The main goal of this study was to conduct an in-field physical ergonomic assessment for hospital staff specialized in cleaning operating rooms according to the subtasks they performed. IMUs were used to record kinematic data from the workers, and they were combined with videotaping for an in-depth analysis. This approach provided a continuous quantification of the risk of hospital workers developing MSDs.

## 2. Methodology

### 2.1. Participants

Eight health workers participated in this experiment (M = 7; F = 1). They were recruited through emails and flyers distributed in the hospital. They had an average age, height, weight and experience of 38.67 ± 13.64 years, 176.38 ± 4.27 cm, 85.33 ± 14.45 kg and 10.80 ± 11.66 years, respectively. Each participant signed an informed consent form. The experimental protocol was approved by the academic ethical committee (CER-Paris-Saclay-2021-293). Inclusion criteria were being a hospital worker who specialized in cleaning operating rooms and with at least one year of experience. The exclusion criterion concerned upper limb injuries within the last 6 months.

### 2.2. Materials

**Figure 1:**
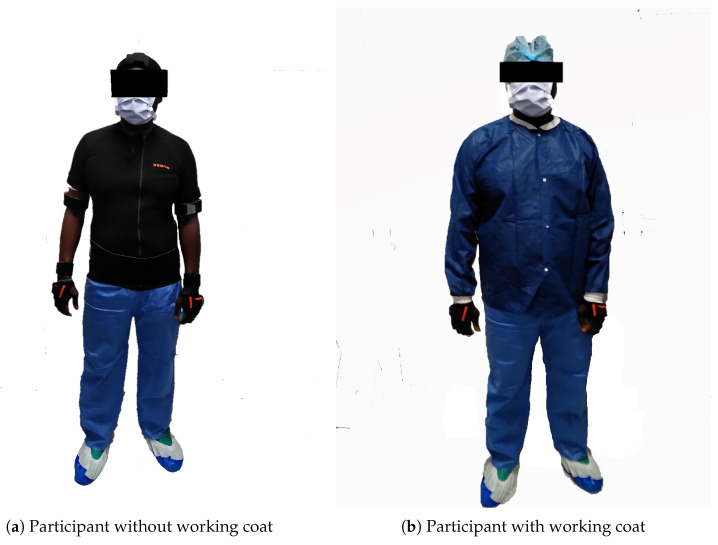
Participant wearing the IMUs system under working clothes.

A wireless IMUs system was used in this study (MTw Awinda, Xsens, Enschede, The Netherlands) to obtain kinematic data of upper body segments. The system was composed of 11 IMUs: one on each shoulder, on each upper arm, on each forearm and on each hand, one on the head, one on the sternum and one on the sacrum (see Figure 1). These IMUs were sampled at 60 Hz [18]. Before each session, a calibration phase was required with the participant wearing the IMUs. During this calibration, the worker had to first stand in an N-pose, i.e., to stand up straight with arms at their sides, for 2 s. Then, he/she had to do a roundtrip walk for 10 s and finally stand in his/her initial position for 2 s again. The aim of the calibration procedure was to estimate segment positions and orientations, which were then applied to the biomechanical model of the participant [19]. This system has been previously validated for a large range of human movements [20]. Concerning ergonomic evaluation, this specific IMU system has been previously considered an accurate and repeatable ergonomic risk assessment tool [21].

In addition to IMUs, a camcorder was used to record movements and subtasks performed by the participant [14]. The camcorder was held by the experimenter so that it was possible to identify and code all subtasks performed by the participant between the beginning and the end of surgical room cleaning without disturbing the workplace and the workers. To synchronize both IMUs and video recordings, the participant had to clap his/her hands at the beginning and the end of the recording session.

The ergonomic assessment was performed through the rapid upper limb assessment (RULA) method, which is an observational method for ergonomic assessment. Based on postures, forces and muscle actions, this tool was developed to evaluate workers’ exposure to risk factors of upper-body MSDs [22]. It has to be noted that the RULA method has been widely employed for ergonomic assessments due to its functionality, both in industrial, health and social assistance environments [23,24].

The automatized RULA method works as follows. Biomechanical data from different upper body segments, mainly joint angles but also some information about position (hand positions, leaning), load and effort, were used to compute local ergonomic scores. These local scores expressed physical constraints applied on specific anatomical areas, i.e., upper arms, lower arms, wrists, neck, trunk and lower limbs. Then, these scores were taken as input arguments in look-up tables to compute three posture scores: one for the upper limb (shoulder, elbow and wrist) and one for the central part (neck, trunk and legs). Two other scores were added to each of these intermediary scores: (i) a muscle-use score computed with respect to the posture held and the repetitiveness of the action performed by the worker, and (ii) a force/load score computed with respect to the load lifted by the worker. Then, the modified intermediary scores for one side and the central part were taken as input arguments in a look-up table to finally compute the global side score. The latter operation was repeated with the modified intermediary scores for the other side and the central part as an input argument to compute the score associated with the other side.

Whether it is local, intermediary or global, the higher a score is, the more likely the worker is at risk of MSDs. Global scores are interpreted as follows:

- 1 or 2 = acceptable posture;
- 3 or 4 = further investigation, change may be needed;
- 5 or 6 = further investigation, change soon;
- 7 = investigate and implement change.

A Nordic musculoskeletal questionnaire was also filled out by each participant before starting the experiment. It is a standardized questionnaire developed to identify MSDs affecting a worker’s upper body. It consists of binary and multiple choice questions about musculoskeletal state that can be answered directly by the involved worker. The questionnaire starts with general questions such as working conditions and physiological information (age, sex, height, weight). Then, it is followed by a summary part, where the worker indicates body zones in which he/she recently suffered from troubles. Finally, the questionnaire is concluded with specific parts with more precise questions about areas in which the worker has already suffered from troubles [25,26,27]. These answers were used to link troubles and injuries to ergonomic scores.

### 2.3. Experimental Procedure

The experiment took place in Pitié-Salpêtrière Hospital (AP-HP, Paris, France), which houses operating rooms for different surgery departments, mainly orthopedic surgery. The participant was first welcomed into a room where he/she was invited to read and sign the informed consent form, answer the Nordic questionnaire and be equipped with the IMUs system (see Figure 1).

When a surgical operation was over, the participant began his/her cleaning job, which consisted of (see Table 1):

- Transferring the patient;
- Cleaning the operating room;
- Tidying up orthopedic materials;
- Cleaning surgery instruments.

Meanwhile, his/her movements were recorded by the IMUs system and the video camera. After the experiment, the participant filled out a feedback comfort questionnaire.

Each session lasted between 40 and 60 min, depending on how long it took to perform the different tasks. All along the experiment, sanitary rules were scrupulously respected.

### 2.4. Data Acquisition and Processing

#### 2.4.1. Ergonomic Scores Computing

Ergonomic scores were computed through a Matlab program that follows the RULA method structure based on conditional statements and look-up tables. To this aim, two kinds of input data were necessary: joint angles computed by the IMUs system, and periods for which a weight was lifted by the participant (identified through video recordings and discussion with the working team). The following joint angles have been obtained: upper arm flexion/extension and internal/external rotation; lower arm flexion/extension; wrist flexion/extension, radioulnar deviation and pronosupination; head flexion/extension, lateral inclination and rotation; trunk flexion/extension, lateral inclination and rotation. Joint angles were expressed relatively to the human body, thus permitting the isolation of each articular degree of freedom. Information about each weight lifted was given by the working team.

Nevertheless, three parameters could not be measured either by IMUs or through video observation: shoulder raising statement, leg score and muscle use score. Based on our direct observations, these parameters were set by default:

- Shoulders were never raised;
- Legs and feet were always supported;
- Posture was never static for more than ten minutes and actions were never repeated four times per minute or more.

#### 2.4.2. Subtasks Segmentation Based on Video Processing

Based on observations, 27 subtasks were identified and segmented with respect to time for each participant. To segment them, the beginning and end of each subtask were first identified through video observations. Then, as video clips and IMUs software were synchronized, it was possible to retrieve kinematical data of each corresponding subtask. These 27 subtasks were classified into 17 groups (see Table 1).

#### 2.4.3. Feature Extraction for Each Subtask

After identifying and segmenting each subtask for each participant, ergonomic features were extracted, i.e., mean scores and percentage of time spent at each range of RULA score (1–2, 3–4, 5–6, 7). Moreover, for local scores, the percentage of time spent at a risky level based on predefined thresholds were computed [14,28]:

- Shoulder and upper arm: 5 (out of a maximum of 6).
- Elbow and lower arm: 2 (out of a maximum of 3).
- Wrist and hand: 5 (out of a maximum of 6).
- Neck and head: 4 (out of a maximum of 6).
- Pelvis and trunk: 4 (out of a maximum of 6).

Finally, mean scores were determined for each subtask in order to perform statistical analyses.

#### 2.4.4. Data Analysis

Statistical tests were performed to determine which subtask induced a high risk of MSDs [29]. Due to the number of participants (n = 8), two non-parametric tests were performed:

- A Friedman test to analyze the effect of subtasks on RULA scores;
- If the Friedman test was significant, a Wilcoxon signed-rank test was performed to identify which subtask was significantly different from the others.

For both tests, independent variables were subtasks, while dependent variables were mean global RULA scores. The significance level was fixed at 0.05. A power calculation was conducted based on a large effect size, a critical α-value of 0.05 and a 1−β of 0.8. To reach a power level of 80%, 12 participants would have been required [30].

## 3. Results

### 3.1. Nordic Musculoskeletal Questionnaire

The Nordic musculoskeletal questionnaire revealed that seven participants were right-handed and one was ambidextrous. Moreover, as summarized in Table 2 and Table 3, these answers also provided information about upper body parts where participants suffered from disorders. It is notable that the lower back was the most frequently affected region, closely followed by the neck.

### 3.2. Global RULA Scores

On average, participants performed their professional activity with a global RULA score of 4.21 ± 1.15 for the right side and 4.19 ± 1.20 for the left side.

**Figure 2:**
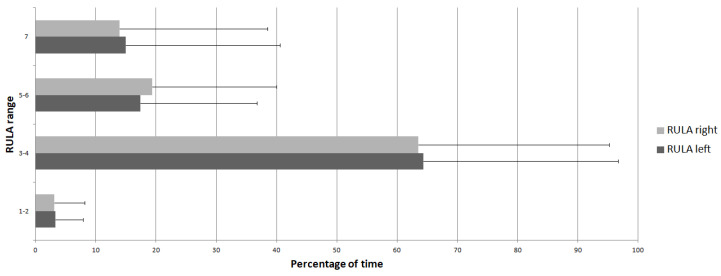
Mean percentage of time spent at each RULA range.

Furthermore, the majority of the time was spent, on average, in the range 3–4, with a percentage of 63.54 ± 31.59% for the right side and 64.33 ± 32.33% for the left side. It was followed by a range of 5–6, with a percentage of 19.38 ± 20.58% for the right side and 17.37 ± 19.34% for the left side. Then, it was range 7 with a percentage of 13.98 ± 24.52% for the right side and 14.97 ± 25.54% for the left side. These two riskiest RULA ranges reached a percentage of 33.36 ± 32.01% for the right side and 32.34 ± 32.04% for the left side when aggregated. Finally, the smallest amount of time was spent, on average, in the range 1–2, with a percentage of 3.09 ± 5.02% for the right side and 3.32 ± 4.52% for the left side. These percentages are plotted in Figure 2.

### 3.3. Local RULA Scores

For each anatomical location, Table 4 presents mean RULA scores and standard deviations. Right and left elbows and lower arms appeared over the risky threshold, as suggested by Vignais et al. [14,28].

**Figure 3:**
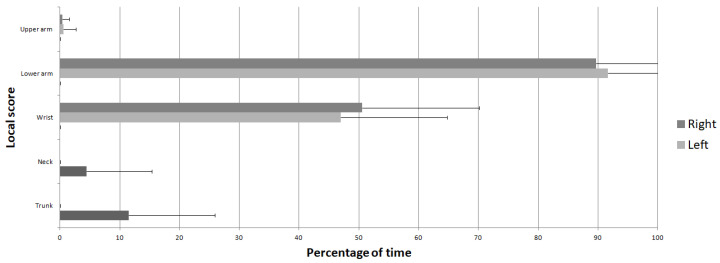
Mean percentage of time spent at a risky level for each segment and articulation.

Furthermore, local scores with the longest duration spent at a risky level were the elbow and lower arm scores, with a percentage of 89.69 ± 27.27% for the right side and 91.70 ± 29.07% for the left side. They were followed by wrists and hands, with a percentage of 50.52 ± 19.56% for the right side and 46.95 ± 17.80% for the left side. Then, the pelvis and trunk areas followed, with a percentage of 11.50 ± 14.40%. Neck and head continued with a percentage of 4.44 ± 10.88%. Finally, the shoulder and upper arm scores represented the smallest amount of time spent at a risky level, with a percentage of 0.38 ± 1.15% for the right side and 0.54 ± 2.08% for the left side. These percentages are plotted in Figure 3.

### 3.4. Subtask Analysis

For each subtask, mean global scores, mean local scores and standard deviations are given in Table 5. Our data suggest that the subtasks associated with the highest global scores were ‘Stretcher moving’ for the right side (6.27 ± 0.28) and ‘Operating table moving’ for the left side (6.36 ± 0.87). The right upper arm reached its highest score during ‘Pressure washing’ (2.07 ± 0.67), while the left upper arm reached it during ‘Handling of lighting’ (2.23 ± 0.39). The riskiest subtasks for the lower arm were ‘Floor cleaning’ and ‘Operating table cleaning’ for the right side (respectively 2.57 ± 0.14 and 2.57 ± 0.25) and ‘Water tanks handling’ for the left side (2.68 ± 0.22). The latter task was also the riskiest one for the right wrist (4.79 ± 0.48), whereas it was ‘Patient transfer’ for the left side (4.84 ± 0.46). Finally, the neck was most exposed during ‘Operating table moving’ (1.69 ± 0.76) and trunk during ‘Operating table cleaning’ (2.96 ± 0.47).

Friedman tests provided significant results when performed on both right ($\chi^{2}$ = 95.098, df = 16, *p* < 0.001) and left ($\chi^{2}$ = 89.108, df = 16, *p* < 0.001) global scores. They were followed by Wilcoxon signed-rank tests to compare each subtask independently. Table 6 summarizes the number of times a subtask was significantly different from the other subtasks, thus highlighting its hazard potential. Subtasks showing the highest numbers of times with a significant difference were ‘Operating table moving’, ‘Stretcher moving’ and ‘Trolley moving’. Other subtasks such as ‘Waste disposal’, ‘Patient transfer’, ‘Operating table disassembly’ and ‘Sheets moving’ were also highlighted.

Finally, mean percentages of time spent at each RULA range for global scores and at a risky level for local scores were given for each subtask in Table 7 and Table 8, respectively. According to a qualitative observation, the subtask that resulted in the most time spent in a RULA value of 7 was ‘Operating table moving’ for both sides (right: 65.66 ± 37.73%; left: 73.67 ± 27.77%). This subtask also resulted in the neck spending most of the time at a risky level (20.89 ± 25.60%), as well as lower arms (right: 99.37 ± 46.93%; left: 99.90 ± 55.12%). ‘Sheets moving’ and ‘Handling of lighting’ resulted in the highest percentage of time at this level for upper arms, respectively, for the right side (1.65 ± 3.36%) and the left side (6.97 ± 5.78%). ‘Water tanks handling’ resulted in the right wrist spending most of the time at this level (64.43 ± 37.55%), whereas it was ‘Patient transfer’ for the left side (69.46 ± 23.19%). Finally, the ‘Floor cleaning’ subtask also induced the highest proportion at this level for trunk (33.47 ± 30.46%).

## 4. Discussion

Due to biomechanical constraints applied to the upper body, hospital cleaners are especially at risk of developing MSDs. This study aimed to conduct a continuous in-field physical ergonomic assessment for hospital staff specialized in cleaning operating rooms. This analysis was performed by taking into account specific subtasks in order to establish precise ergonomic recommendations. Assessments were based on a system combining an IMU-based RULA method and video recordings. Kinematic data were calculated from IMUs, while lifted weights were informed by the hospital staff. Then, these data were processed to compute RULA scores with their features: for global scores, mean values and percentages of time spent in each RULA range; for local ones, mean values and percentages of time spent at a risky level. Subtasks were identified and segmented through video analysis, and then each of them could be associated with the above-mentioned RULA features.

Results showed that average global RULA scores were closer to the range 3–4. This range was also the one in which the biggest amount of time was spent on average. Thus, the average activity performed by health workers might need further investigation, and change might be needed [22]. This study can be compared to a work that used a similar methodology on novice workers performing industrial manual tasks [28]. In the latter study, some workers had access to RULA feedback in real-time, while others did not. Outcomes from the current study appear to be in the same range as those from participants without RULA feedback (right side: 4.4 ± 0.65; left side: 4.31 ± 0.46). The same observation can be made for the range on which the biggest part of time is spent on average. In another study with a similar methodology applied to workers preparing biomedical devices [14], higher mean values were obtained (right side: 6 ± 0.87; left side: 6.2 ± 0.78) with a majority of time spent on the range 7 (right side: 49.19 ± 35.27%; left side: 55.5 ± 29.69%). In the latter study, participants were sitting on a chair with subsequently less moving space around the workplace, which can explain the differences with results from the current study.

Local RULA scores have been computed in order to identify upper body areas that were more at risk during the occupational activity. The elbow and lower arm mean values were higher than the predefined thresholds of risky levels. Moreover, according to mean percentages of time spent at this level, these segments, as well as wrists and hands, appeared to be more frequently required to adopt hazardous postures compared to other segments/joints. This result differs from current data about the risk of MSDs in hospital workers, as work-related MSDs usually highlight the lower back in nurses [1,5,6]. However, MSDs among nursing assistants affect both their back and their upper limbs, elbows, lower arms, wrists and hands, according to a qualitative study [7]. In the current study, the impact of work on the lumbar area was highlighted by the Nordic musculoskeletal questionnaire. Indeed, lower back and neck were the most frequently reported affected regions. This paradoxical outcome, i.e., subjective vs. objective results, may be explained by the fact that pathomechanical links exist between upper limb exposure and the appearance of MSDs at the back level [31,32,33].

The subtask analysis was performed to identify specific tasks that induced awkward postures. Those subtasks associated with the highest global scores were ‘Stretcher moving’ and ‘Operating table moving’. The latter was also associated with the highest percentage of time spent at a RULA value of 7. Moreover, with ‘Trolley moving’, they were the three subtasks whose average scores were higher than 6. Logically, these subtasks were the most frequently significant compared to the others in terms of global RULA scores. It has to be noted that the current methodology permitted the identification of these risky subtasks by combining objective biomechanical data from IMUs and accurate activity coding from video recordings. On the contrary, previous studies performed on hospital staff used analyses reduced to specific tasks [1,8,15]. Among subtasks identified in the current study, ‘Operating table moving’ had the highest average neck score and percentage of time at a risky level. It was likely influenced by the force/load score since this subtask involved displacements of heavy objects. Therefore, it may be relevant to find a way to reduce these loads, for example, through motorized assistance [34].

Focusing on the riskiest upper body areas, subtasks with the highest elbow and lower arm mean scores were ‘Floor cleaning’, ‘Operating table cleaning’ and ‘Water tanks handling’. Subtasks with the highest wrist and hand mean scores were ‘Water tanks handling’ and ‘Patient transfer’. As for subjective riskiest areas identified by the Nordic musculoskeletal questionnaire, the neck was most at risk during ‘Operating table moving’, while the trunk was most exposed during ‘Operating table cleaning’ (mean value) and during ‘Floor cleaning’ (percentage of time at risk). Except for ‘Patient transfer’, all these risky subtasks involved the manipulation of devices, which may be improved through specific ergonomic interventions in material handling operations [35]. The use of an assistive device, e.g., exoskeleton, might also be considered to decrease the exposure to MSDs [36,37]. Finally, concerning subtasks such as ‘Patient transfer’, a training program might be relevant to decrease the risk of MSDs [38].

The whole subtask analysis permitted the highlighting of specific musculoskeletal risks for hospital cleaners. Some of these subtasks involved the displacement of an object, e.g., ‘Stretcher moving’, ‘Operating table moving’, ‘Trolley moving’. For that kind of activity, elbows and lower arms were particularly at risk. Redesigning rolling equipment by considering ergonomic aspects of pulling/pushing actions may help reduce these risks [39,40]. Concerning typical cleaning activities, like ‘Floor cleaning’ and ‘Operating table cleaning’, elbows and lower arms were more frequently required. Involving cleaners in a redesign process of the cleaning hand tools based on ease-of-use and comfort has been shown to significantly improve the quality of work [41,42]. Finally, transferring the patient from the stretcher to the operating room was also extremely demanding, as previously shown in the literature [43]. Highly detailed recommendations have thus been expressed for the operating team to reduce the physical workload and decrease the risk of MSDs, including the use of transfer devices and intelligent beds [44].

## 5. Limitations

The first limitation related to the current study came from the proposed methodology, which was not able to collect all the necessary information for the RULA ergonomic assessment. More precisely, the shoulder raising statement, leg score and muscle use score were set by default according to preliminary observations. The limit on leg score could be overcome in the future by placing supplemental IMUs on lower limbs.

Secondly, the number of participants may have limited the ergonomic scope of this study. However, it is often difficult to recruit a large number of participants in a specific working environment like the hospital sector. Thus, it would be relevant to apply this methodology to a higher number of participants (for example, workers from several hospitals).

Thirdly, the definition of subtasks may be questioned. Indeed, they have been defined empirically after video observation and discussion with hospital cleaners for their meaningfulness. However, some methods allow the worker’s operation to be expressed into simple motion elements, such as Therbligs [45,46]. This methodology might be used in future studies to better describe subtasks and facilitate the comparison with ergonomic data from the literature.

Finally, the continuous ergonomic assessment performed in the current study was based on the RULA table [22]. This method considers the following risk factors: posture, muscle use, weight of load, shock, task duration and repetitiveness. More precisely, this method attempts to quantify the combination of these risk factors with the aim of obtaining an overall exposure score. Nevertheless, there is a lack of epidemiological data supporting the suggested patterns [13]. Although it is one of the most cited methods in the ergonomic literature [24], and it is frequently applied in industry [47], only comparative studies are permitted to evaluate its relevance. Thus, it appears necessary to further investigate the relevance of the RULA method in view of recent methods based on discomfort and epidemiological data [48]. Moreover, the RULA method was initially developed for static postures. Therefore, the cumulative aspect of MSDs appearance, as pointed out in previous studies [14,49], was not taken into account.

## 6. Conclusions

The goal of this study was to conduct an in-field physical ergonomic assessment for hospital workers dedicated to cleaning operating rooms. The risk of developing upper body work-related MSDs was quantified through a methodology combining IMU-based observational methods and video recordings. This permitted the identification of subtasks causing the highest risk for workers. This methodology might, therefore, be tested on a larger population of participants to support current outcomes. Moreover, inspired by recent works [50,51], a machine learning-based system could be trained and tested on specific workers to automatically identify risky subtasks. Given the societal burden of MSDs affecting hospital workers, the use of advanced aiding systems and assistive devices would also be valuable. Finally, the proposed methodology may permit the evaluation of these suggested ergonomic interventions (pre/post effects). 

## Figures and Tables

**Table 1:** Description of the different groups of subtasks and the subtasks.

Group of Subtasks	Subtask
Waste disposal	Waste pickup
	Garbage disposal
Handling of lighting	Lighting protections removal
	Lighting switching off
	Lighting cleaning
	Radiography device handling
Handling around the patient	Patient unequipping
	Patient equipping
	Patient holding
	Installing patient on stretcher
Cables and pipes handling	Pipe disconnection
	Cables untangling
Patient transfer	Patient transfer
Surfaces and tools cleaning	Equipments cleaning
	Sink cleaning
Various objects moving	Various objects moving
	Water disinfection
Floor cleaning	Floor cleaning
Box lifting	Box lifting
Water tanks handling	Water tanks handling
Pressure washing	Pressure washing
Operating table moving	Operating table moving
Stretcher moving	Stretcher moving
Operating table cleaning	Operating table cleaning
Operating table disassembly	Operating table disassembly
Sheets moving	Sheets moving
Trolley moving	Trolley moving

**Table 2:** Frequencies of upper body work-related MSDs over the last 12 months and the last 7 days and work limitations. ‘Limitation in the workday’ corresponds to the percentage of participants who reported a reduction in daily activities due to anatomical constraints.

Body Region	Last 12 Months	Last 7 Days	Limitation in the Workday
Neck	55.5%	22.2%	22.2%
Shoulders	44.4%	11.1%	0%
Elbows	11.1%	0%	0%
Wrists	44.4%	0%	0%
Upper back	33.3%	0%	11.1%
Lower back	66.7%	33.3%	44.4%

**Table 3:** Frequencies of upper body work-related MSDs in the past, injuries and work limitations.

Body Region	Disorders	Injuries	Need to Change Jobs
Neck	66.7%	11.1%	0%
Shoulders	66.7%	0%	0%
Elbows	11.1%	0%	0%
Wrists	44.4%	22.2%	0%
Upper back	44.4%	0%	22.2%
Lower back	77.8%	22.2%	11.1%

**Table 4:** Mean local RULA scores and standard deviations.

Location	Mean Local RULA Score
Right shoulder and upper arm	1.75 ± 0.39
Left shoulder and upper arm	1.68 ± 0.34
Right elbow and lower arm	2.33 ± 0.30
Left elbow and lower arm	2.37 ± 0.28
Right wrist and hand	4.50 ± 0.37
Left wrist and hand	4.41 ± 0.40
Pelvis and trunk	2.41 ± 0.70
Neck and head	1.29 ± 0.36

**Table 5:** Mean global and local RULA scores and standard deviations for each subtask. Bold numbers represent the highest mean values per column.

	Global Score		Upper Arm Score		Lower Arm Score		Wrist Score		Neck Score	Trunk Score
	Right	Left	Right	Left	Right	Left	Right	Left		
Waste disposal	3.31 ± 0.19	3.27 ± 0.15	1.64 ± 0.26	1.49 ± 0.16	2.34 ± 0.05	2.43 ± 0.20	4.30 ± 0.33	4.23 ± 0.21	1.18 ± 0.09	2.45 ± 0.46
Handling of lighting	3.65 ± 0.48	3.76 ± 0.54	2.05 ± 0.32	**2.23** ± **0.39**	2.10 ± 0.28	2.18 ± 0.23	4.53 ± 0.39	4.34 ± 0.26	1.62 ± 0.45	2.13 ± 0.72
Handling around the patient	3.53 ± 0.38	3.47 ± 0.38	1.75 ± 0.37	1.63 ± 0.16	2.33 ± 0.38	2.30 ± 0.26	4.49 ± 0.35	4.47 ± 0.42	1.30 ± 0.28	2.12 ± 068
Cables and pipes handling	3.42 ± 0.17	3.37 ± 0.21	1.73 ± 0.40	1.75 ± 0.27	2.16 ± 0.35	2.20 ± 0.24	4.41 ± 0.27	4.32 ± 0.27	1.19 ± 0.16	2.73 ± 0.69
Patient transfer	4.95 ± 1.05	4.98 ± 1.12	1.70 ± 0.48	1.66 ± 0.30	2.51 ± 0.36	2.28 ± 0.29	4.78 ± 0.38	**4.84** ± **0.46**	1.55 ± 0.89	2.04 ± 0.80
Surfaces and tools cleaning	3.61 ± 0.32	3.58 ± 0.41	1.67 ± 0.27	1.66 ± 0.23	2.31 ± 0.23	2.31 ± 0.35	4.32 ± 0.27	4.23 ± 0.18	1.33 ± 0.20	2.35 ± 0.64
Various objects moving	3.85 ± 0.50	3.81 ± 0.57	1.63 ± 0.24	1.51 ± 0.15	2.31 ± 0.21	2.32 ± 0.26	4.29 ± 0.21	4.18 ± 0.41	1.31 ± 0.19	2.36 ± 0.79
Floor cleaning	3.80 ± 0.53	3.79 ± 0.57	1.96 ± 0.46	1.69 ± 0.24	**2.57** ± **0.14**	2.50 ± 0.10	4.64 ± 0.29	4.70 ± 0.40	1.13 ± 0.13	2.95 ± 0.72
Box lifting	5.35 ± 1.45	5.33 ± 1.59	1.70 ± 0.53	1.71 ± 0.44	2.36 ± 0.18	2.41 ± 0.15	4.56 ± 0.32	4.48 ± 0.35	1.30 ± 0.27	2.32 ± 0.76
Water tanks handling	3.35 ± 0.55	3.62 ± 0.51	1.61 ± 0.30	1.93 ± 0.57	2.27 ± 0.36	**2.68** ± **0.22**	**4.79** ± **0.48**	4.69 ± 0.22	1.31 ± 0.34	2.63 ± 0.73
Pressure washing	3.88 ± 0.44	3.68 ± 0.50	**2.07** ± **0.67**	1.66 ± 0.20	1.94 ± 0.53	2.24 ± 0.38	4.65 ± 0.64	4.40 ± 0.30	1.38 ± 0.42	2.38 ± 0.83
Operating table moving	6.24 ± 0.95	**6.36** ± **0.87**	1.56 ± 0.43	1.72 ± 0.33	2.44 ± 0.34	2.52 ± 0.39	4.25 ± 0.39	4.23 ± 0.69	**1.69** ± **0.76**	2.88 ± 0.50
Stretcher moving	**6.27** ± **0.28**	6.30 ± 0.33	1.63 ± 0.27	1.54 ± 0.36	2.33 ± 0.20	2.51 ± 0.28	4.57 ± 0.48	4.59 ± 0.46	1.10 ± 0.10	1.89 ± 0.58
Operating table cleaning	3.80 ± 0.54	3.56 ± 0.52	1.72 ± 0.24	1.54 ± 0.39	**2.57** ± **0.25**	2.43 ± 0.31	4.66 ± 0.30	4.17 ± 0.42	1.31 ± 0.22	**2.96** ± **0.47**
Operating table disassembly	4.48 ± 0.56	4.47 ± 0.68	1.73 ± 0.25	1.70 ± 0.32	2.21 ± 0.33	2.37 ± 0.12	4.47 ± 0.30	4.34 ± 0.30	1.17 ± 0.22	2.65 ± 0.55
Sheets moving	3.27 ± 0.29	3.18 ± 0.22	1.80 ± 0.56	1.68 ± 0.51	2.23 ± 0.16	2.16 ± 0.42	4.40 ± 0.39	4.31 ± 0.36	1.11 ± 0.09	2.15 ± 0.66
Trolley moving	6.14 ± 0.74	6.18 ± 0.83	1.71 ± 0.45	1.71 ± 0.34	2.55 ± 0.20	2.63 ± 0.14	4.47 ± 0.20	4.48 ± 0.25	1.19 ± 0.08	2.34 ± 0.80

**Table 6:** Number of subtasks with which each subtask has significant differences.

Subtask	Number of Significant Differences	
	Right	Left
Waste disposal	10	7
Handling of lighting	5	4
Handling around the patient	6	5
Cables and pipes handling	6	4
Patient transfer	10	11
Surfaces and tools cleaning	5	5
Various objects moving	5	6
Floor cleaning	5	6
Box lifting	6	3
Water tanks handling	6	3
Pressure washing	5	5
Operating table moving	13	13
Stretcher moving	13	13
Operating table cleaning	5	5
Operating table disassembly	11	8
Sheets moving	9	10
Trolley moving	13	12

**Table 7:** Mean percentage of time spent at each RULA range per subtask. Bold numbers represent the highest mean values.

	Percentage of Time Spent in Each RULA Range							
	Right				Left			
	**1–2**	**3–4**	**5–6**	**7**	1–2	**3–4**	**5–6**	**7**
Waste disposal	4.93 ± 5.01	86.16 ± 5.70	8.77 ± 5.27	0.14 ± 0.16	5.55 ± 3.92	86.49 ± 6.61	7.82 ± 5.14	0.14 ± 0.14
Handling of lighting	7.57 ± 7.15	73.81 ± 13.44	15.55 ± 14.16	3.06 ± 4.84	5.39 ± 4.15	72.56 ± 14.42	15.74 ± 11.91	6.31 ± 6.14
Handling around the patient	3.89 ± 4.36	83.02 ± 14.77	9.45 ± 8.92	3.64 ± 4.57	4.14 ± 3.66	83.55 ± 15.11	10.22 ± 11.18	2.09 ± 2.46
Cables and pipes handling	3.40 ± 4.55	86.77 ± 10.74	9.83 ± 7.05	0	1.50 ± 1.84	91.54 ± 4.78	6.56 ± 4.75	0.41 ± 0.64
Patient transfer	0.25 ± 0.44	44.39 ± 35.45	30.19 ± 30.42	25.17 ± 25.32	1.23 ± 2.09	42.69 ± 36.65	31.52 ± 30.08	24.56 ± 22.79
Surfaces and tools cleaning	3.46 ± 4.46	81.34 ± 10.03	12.35 ± 8.29	2.85 ± 3.07	4.52 ± 5.37	81.08 ± 10.17	11.14 ± 7.81	3.26 ± 4.66
Various objects moving	3.84 ± 5.75	73.62 ± 12.63	14.20 ± 5.41	8.34 ± 9.49	4.11 ± 4.90	73.47 ± 14.60	14.32 ± 7.01	8.11 ± 9.83
Floor cleaning	1.14 ± 2.05	76.10 ± 20.08	22.60 ± 21.10	0.16 ± 0.17	2.48 ± 3.78	74.36 ± 17.33	22.96 ± 19.74	0.19 ± 0.27
Box lifting	0.22 ± 0.55	37.32 ± 45.34	27.77 ± 33.71	34.69 ± 37.37	0.28 ± 0.66	37.15 ± 45.58	23.85 ± 27.04	38.72 ± 37.14
Water tanks handling	7.45 ± 13.50	80.01 ± 11.23	12.53 ± 13.53	0.01 ± 0.02	1.65 ± 2.22	85.88 ± 11.91	12.36 ± 13.58	0.11 ± 0.22
Pressure washing	1.43 ± 3.01	76.70 ± 16.60	17.97 ± 14.25	3.90 ± 6.02	3.75 ± 6.37	76.35 ± 14.77	16.88 ± 12.33	3.02 ± 4.29
Operating table moving	2.82 ± 5.63	10.48 ± 19.16	21.05 ± 39.37	**65.66** ± **37.73**	0.07 ± 0.14	12.79 ± 23.45	13.47 ± 24.52	**73.67** ± **27.77**
Stretcher moving	0.01 ± 0.02	5.70 ± 8.38	49.67 ± 25.96	44.62 ± 22.37	0.06 ± 0.15	5.95 ± 8.42	45.46 ± 33.15	48.53 ± 29.52
Operating table cleaning	3.23 ± 4.30	72.38 ± 15.88	22.14 ± 13.41	2.26 ± 5.54	4.98 ± 6.22	82.53 ± 17.03	10.05 ± 10.44	2.44 ± 5.98
Operating table disassembly	2.23 ± 3.23	56.28 ± 17.22	22.93 ± 14.80	18.56 ± 19.72	3.86 ± 4.12	56.65 ± 17.80	18.23 ± 14.47	21.26 ± 16.99
Sheets moving	6.55 ± 6.05	85.93 ± 6.14	7.20 ± 7.48	0.31 ± 0.58	8.63 ± 7.49	86.19 ± 8.49	4.96 ± 5.86	0.22 ± 0.58
Trolley moving	0.33 ± 0.72	14.00 ± 20.36	30.84 ± 31.39	54.83 ± 29.51	0.35 ± 0.52	12.60 ± 22.27	31.49 ± 28.02	55.57 ± 30.74

**Table 8:** Mean percentage of time spent at a risky level for each local RULA score per subtask. Bold numbers represent the highest mean values.

	Percentage of Time Spent at a Risky Level for Each Local RULA Score							
	Upper Arm Score		Lower Arm Score		Wrist Score		Neck Score	Trunk Score
	Right	Left	Right	Left	Right	Left		
Waste disposal	0.32 ± 0.59	0.35 ± 0.72	91.63 ± 4.12	94.23 ± 20.63	41.53 ± 12.67	39.59 ± 9.50	2.96 ± 3.02	9.39 ± 6.58
Handling of lighting	1.33 ± 1.63	**6.97** ± **5.78**	80.11 ± 14.10	85.64 ± 19.00	53.48 ± 16.97	45.25 ± 13.10	16.96 ± 16.36	6.64 ± 11.61
Handling around the patient	0.33 ± 0.56	0	88.27 ± 30.59	92.48 ± 28.53	50.42 ± 21.01	46.97 ± 20.37	3.45 ± 4.56	5.29 ± 5.76
Cables and pipes handling	0.69 ± 1.47	0.07 ± 0.13	82.20 ± 26.51	83.30 ± 22.29	47.09 ± 14.82	40.61 ± 9.61	0.83 ± 1.14	13.60 ± 8.64
Patient transfer	0.44 ± 1.17	0	96.08 ± 41.19	94.45 ± 33.40	64.42 ± 25.64	**69.46** ± **23.19**	16.07 ± 27.55	2.08 ± 3.56
Surfaces and tools cleaning	0.05 ± 0.14	0.47 ± 0.98	89.68 ± 23.94	86.43 ± 33.18	39.63 ± 9.85	39.02 ± 11.01	2.05 ± 2.39	8.33 ± 6.88
Various objects moving	0.47 ± 0.98	0.09 ± 0.18	87.64 ± 15.91	88.04 ± 17.88	39.72 ± 9.50	37.89 ± 14.70	3.58 ± 5.50	9.46 ± 9.48
Floor cleaning	0.26 ± 0.66	0.14 ± 0.20	93.94 ± 12.71	96.78 ± 11.44	57.70 ± 12.22	59.83 ± 21.94	0.28 ± 0.37	**33.47** ± **30.46**
Box lifting	0.05 ± 0.12	0.06 ± 0.14	93.83 ± 18.37	94.93 ± 24.15	56.76 ± 17.84	51.53 ± 21.41	3.39 ± 5.98	8.23 ± 9.42
Water tanks handling	0.25 ± 0.49	0.56 ± 1.12	90.11 ± 47.19	94.75 ± 18.45	**64.43** ± **37.55**	53.52 ± 21.25	0.52 ± 1.05	13.46 ± 12.18
Pressure washing	0	0	67.87 ± 26.13	93.30 ± 45.18	53.89 ± 33.84	47.56 ± 9.69	0.02 ± 0.05	11.29 ± 17.60
Operating table moving	0	0.38 ± 0.76	**99.37** ± **46.93**	**99.90** ± **55.12**	46.67 ± 26.51	39.72 ± 20.98	**20.89** ± **25.60**	11.93 ± 8.63
Stretcher moving	0	0	96.54 ± 33.01	96.74 ± 35.92	53.23 ± 23.46	52.98 ± 23.20	0.22 ± 0.37	3.36 ± 4.69
Operating table cleaning	0	0.14 ± 0.35	94.42 ± 26.28	91.02 ± 28.12	55.64 ± 18.69	36.06 ± 17.41	2.66 ± 6.53	25.99 ± 17.11
Operating table disassembly	0.34 ± 0.59	0	85.13 ± 25.39	92.45 ± 14.13	51.44 ± 12.93	45.57 ± 12.92	2.44 ± 4.41	18.50 ± 12.53
Sheets moving	**1.65** ± **3.36**	0.25 ± 0.57	87.57 ± 11.33	80.00 ± 35.58	42.80 ± 18.25	43.13 ± 18.21	0.76 ± 0.96	7.01 ± 8.76
Trolley moving	0	0.06 ± 0.13	98.40 ± 26.76	99.32 ± 19.15	47.91 ± 14.92	47.75 ± 15.01	2.32 ± 2.66	6.55 ± 10.05

## Data Availability

The raw data supporting the conclusions of this article will be made available by the authors on request.
